# Supplementary material for: Comparing healthcare systems between the Netherlands and Australia in management for children with acute gastroenteritis
Source: PLoS One. 2024 Jul 24;19(7):e0306739. doi: 10.1371/journal.pone.0306739 (PMC11268636; doi:10.1371/journal.pone.0306739)
Supplement: S1 File — (DOCX) [file pone.0306739.s001.docx]

**S1.1 Appendix** Questionnaire public health (outbreak management)

**Comparing healthcare systems: acute gastroenteritis outbreaks in children**This questionnaire focusses on acute infectious gastroenteritis and outbreaks of this disease in children on a public health level in the State of Victoria/Netherlands. Some questions may have more answers, please provide as much information as you have.

**Governance**Policy and vision

- What is the public health policy^1^ and vision of the State of Victoria/Netherlands for management of acute gastroenteritis outbreaks in children with goals and targets? Can you provide documents or websites?
- Does the public health policy of the State of Victoria/Netherlands include a multisectoral approach^2^ for acute gastroenteritis outbreaks in children? If yes, how?
- Are there recommendations and transmural agreements for acute gastroenteritis outbreaks in the State of Victoria/Netherlands (across different health units and providers)? If yes, can you explain this?

Stakeholder voice

- Who is responsible for the development and review of the public health policy for management of acute gastroenteritis outbreaks in children?
- Do/have stakeholders participate(d) in the development and review of the public health policy for the management of acute gastroenteritis outbreaks in children? If yes, which stakeholders are/were involved and in which way?
- What mechanisms or rules are in place to ensure involvement of stakeholders in the development and review of this public health policy?

Information and intelligence

- Is there a regular monitoring and evaluation for acute gastroenteritis outbreaks in children in the State of Victoria/Netherlands? If yes, how?
- What data on acute gastroenteritis outbreaks is the government of the State of Victoria/Netherlands committed to collect for decision-making?
- Are relevant databases available for acute gastroenteritis outbreaks (i.e., registration, health insurance, pharmaceutical databases, health facility reporting and resource tracking systems)? If yes, how are these connected and can you provide documents or websites?
- How is data sharing regarding acute gastroenteritis outbreaks arranged between various layers of government and stakeholders?

Legislation and regulation

- Which legislation is applicable for acute gastroenteritis outbreaks in children?
- Are existing health laws aligned with the public health policy for acute gastroenteritis outbreaks? If yes, can you explain this and provide documents or websites?
- Is there a legislation that affects the prevention of children with acute gastroenteritis (i.e., vaccination, staying at home)? If yes, can you explain this and provide documents or websites?

**Resource generation**Health workforce

- Which organizations and healthcare professionals are involved in the implementation of the public health policy regarding acute gastroenteritis outbreaks?
- Are there enough healthcare professionals available for the workforce in acute gastroenteritis outbreaks? Can you provide evidence for this?
- Do healthcare professionals receive specific training for the management of acute gastroenteritis outbreaks (i.e., medicine study, outbreak strategies)? If yes, what training is offered? Can you provide specific websites or documents about this training?

Infrastructure and medical equipment

- What basic infrastructure and medical equipment is available for acute gastroenteritis outbreaks (i.e., health facilities, information systems, electronic files, additional testing)? Can you provide evidence about the quality and quantity of it?
- How is the infrastructure and medical equipment distributed across different types of care for acute gastroenteritis outbreaks (i.e., primary, secondary)? And in different sectors (i.e., private, public)?

Pharmaceuticals and other consumables

- Which pharmaceuticals and other consumables are available for acute gastroenteritis outbreaks (i.e., vaccines, oral rehydration solutions, antibiotics, anti-emetics, antipyretics)? Can you comment on the quantity and/or availability of it?
- Who is responsible for providing these pharmaceuticals and other consumables to healthcare professionals?
- How are pharmaceuticals distributed across different types of care for acute gastroenteritis outbreaks (i.e., primary, secondary)? And in different sectors (i.e., private, public)?

**Financing**

- How is the management of acute gastroenteritis outbreaks, including vaccination, financed?
- How are the pharmaceuticals and other consumables for acute gastroenteritis outbreaks financed (i.e., government, health insurers, consumers)?

**Service delivery**

- Which healthcare professionals are involved in the management of acute gastroenteritis outbreaks (i.e., infectious disease physicians, nurses, general practitioners, triage specialists, paediatricians)?
- Could you describe how the access to care in acute gastroenteritis outbreaks is organized (i.e., telephonic contact, home visit, regular hours, out-of-hours, emergency, primary and secondary care)?
- How is the service delivery arranged in acute gastroenteritis outbreaks (i.e., information, prescription of pharmaceuticals, referrals, vaccination)?

^1^Public health policy plays an essential role in defining a country’s vision, policy directions and strategies for ensuring the health of its population (WHO).
^2^Multisectoal approach refers to deliberate collaboration among various stakeholder groups (e.g., government, civil society, and private sector) and sectors (e.g., health, environment, and economy) to jointly achieve a policy outcome. (Salunke, et al. Multi sectoral approach for promoting public health).

**S1.2 Appendix** Questionnaire clinical daily care

**Comparing healthcare systems: clinical daily care in children with acute gastroenteritis**This questionnaire focusses on clinical daily care in children with acute infectious gastroenteritis in the State of Victoria/Netherlands. Some questions may have more answers, please provide as much information as you have.

**Clinical care**

- Could you describe how the clinical care for children with acute gastroenteritis is organized in the State of Victoria/Netherlands (i.e., telephonic contact, home visit, emergency, primary and secondary care)?
- Could you describe how the access to care for children with acute gastroenteritis is organized in the State of Victoria/Netherlands (i.e., regular hours, out-of-hours, primary and secondary care)?
- Which healthcare professionals are involved in the clinical care for children with acute gastroenteritis (e.g., triage specialists, nurses, general practitioners, paediatricians)?
- Who is responsible for delivering services and/or pharmaceuticals to children with acute gastroenteritis (e.g., advice, information, referrals, admission, additional testing)?

**Resources**

Infrastructure and medical equipment

- What basic infrastructure is available for the clinical care of children with acute gastroenteritis (i.e., health facilities)? Can you comment on the quantity and/or availability of it?
- What medical equipment is available for the clinical care of children with acute gastroenteritis (i.e., electronic files, additional testing)? Can you comment on the quantity and/or availability of it?
- How is the infrastructure and medical equipment distributed across different types of care for children with acute gastroenteritis (i.e., primary, secondary)? And in different sectors (i.e., private, public)?

Pharmaceuticals and other consumables

- Which pharmaceuticals and other consumables are available for the clinical care in children with acute gastroenteritis (i.e., apple juice, oral rehydration solutions, antibiotics, anti-emetics, antipyretics)? Can you comment on the quantity and/or availability of it?
- Who is responsible for providing these pharmaceuticals and other consumables to healthcare professionals?
- How are pharmaceuticals distributed across different types of care for the clinical care in children with acute gastroenteritis (i.e., primary, secondary)? And in different sectors (i.e., private, public)?

Human resources

- Do healthcare professionals receive specific training for the clinical care of children with acute gastroenteritis (i.e., medicine study, primary care course, triage course)? If yes, what training and can you provide specific websites or documents about this training?
- Are there enough healthcare professionals available for the workforce in the clinical care of children with acute gastroenteritis? Can you provide evidence for this?

Information

- Is there a guideline for healthcare professionals for the clinical care in children with acute gastroenteritis? If yes, can you provide documents or websites?
- Who is responsible for the development and review of this guideline?
- Is there any information/education available for parents of children with acute gastroenteritis? If yes, can you provide documents or websites?

**Financing**

- How are healthcare providers paid for delivering services to children with acute gastroenteritis?
- How is the clinical care of children with acute gastroenteritis financed (i.e., patients, health insurers, national funds/government)?
- How are the pharmaceuticals and other consumables for the clinical care in children with acute gastroenteritis financed?
- Is there an authority overseeing the financing for delivering the clinical care in children with acute gastroenteritis?

**Government**

- What is the role of the government in the clinical care for children with acute gastroenteritis (i.e., development and implementation)?
- Which ministries are involved in the clinical care for children with acute gastroenteritis?
- Who is responsible for overseeing the standard of clinical care of healthcare professionals involved in the clinical care of children with acute gastroenteritis?
- Which legislation is applicable for the clinical care in children with acute gastroenteritis?
