# Supplementary material for: Comparing healthcare systems between the Netherlands and Australia in management for children with acute gastroenteritis
Source: PLoS One. 2024 Jul 24;19(7):e0306739. doi: 10.1371/journal.pone.0306739 (PMC11268636; doi:10.1371/journal.pone.0306739)
Supplement: Attachment — Submitted filename: Response to Reviewers 2.0 .docx [file pone.0306739.s004.docx]

**Response to Reviewers 2.0**

We would like to thank the editor for the opportunity to submit a revised version of our manuscript (PONE-D-23-40570). We thank the reviewers for the constructive comments and suggestions to improve our manuscript. The detailed response to each of their comments is provided in this document. We hope our revisions will meet the expectations put forward by the reviewers. The page and line numbers we refer to correspond to the ‘track changes’ document.

**Reviewer #1: All my suggestions have been adequately addressed. The final manuscript reads well and my congratulations to the submitting authors.**

Thank you very much.

**Reviewer #2: The edits made by the authors has substantially improved the paper, and I commend the authors in their efforts. The aim of the paper and how this analysis sets out to address this is much clearer.**

**Some final minor points for attention.**

1. **I note the authors have mentioned rotavirus vaccination and specific therapies (eg ondansetron) without reference as to why these are relevant to childhood gastroenteritis. For unfamiliar readers, I would suggest briefly noting why it is relevant at first mention (this is described much later in the paper) or give a very brief overview of current best public health and clinical care practice for the prevention and management of childhood gastroenteritis in the introduction.**

Thank you for the comment. We added the sentence: ‘Acute gastroenteritis is a highly contagious disease with rotavirus being the most common cause (2, 23)’ (Page 10, line 162).

We added the word ‘an antiemetic medicine’ after ondansetron to give clarity (Page 16, line 303).

In addition, we added it being an anti-emetic in the abstract.

2. **Table 1 is terrific and really helps with orienting the reader as to what is coming in the results. This needs a footnote to explain the ‘no jab, no pay’ policy.**

Thank you for the compliment. We have added in the footnote of the table the following: ‘1No Jab No Pay: to access family assistance payments, immunisation is required. No Jab No Play: to attend childcare, immunisation is required.’ (Page 9, Table 1). In addition, because of the above comment, we added a footnote about ondansetron too.

3. **The sentence ‘The methods of this study were based on a previous performed advisory report comparing health’ needs review for grammatical correctness. Also, with respect to this could the authors please briefly explain why this method was appropriate to this study aim, and a brief overview of what was involved.**

We have amended the sentence thus: The methods of this study were based on those used in a prior report comparing health systems between different countries (12).

4. **In the methods and under study limitations, assuming no differences across jurisdictions is unreasonable; each will have differing policies, funding arrangements, delivery of services – albeit quite likely minor. This should be amended in the paper.**

Everything that is stated in the manuscript is applicable for each jurisdiction. We added ‘The primary care system and National immunisation program funding and policies are run by the Commonwealth and do not vary in application by jurisdiction.’ To the limitations (Page 21, line 401-403)

5. **Re the sentence “Citizens can choose to purchase extra private health insurance to access additional healthcare services (largely hospital care) and benefits not covered by Medicare.” The reference to ‘and benefits’ is vague and adds little. I suggest deleting this.**

We deleted the ‘and benefits’ (Page 11, line 189).

6. **I would suggest delete “, often leading GPs to charge higher fees’ from the following sentence for clarity, and ‘amend resulting in’ to ‘and has been associated with’ to avoid inferring causality.**

We deleted the ‘often leading GPs to charge higher fee’ (Page 12, line 213, 214) and reformulated ‘resulting in’ to ‘has been associated with’ (page 12, line 214).

7. **“However, the remuneration has not been adjusted for over ten years for inflation and rising costs, often leading GPs to charge higher fees, resulting in increased co-payments and out-of-pocket costs for patients. Some GPs offer ‘bulk-billing’, where Medicare covers the full consultation cost, and GPs bill Medicare directly instead of patients.”**

**In addition, GP’s accept payment directly from Medicare on behalf of the patient, rather than billing Medicare directly. The text should be amended to reflect this.**

Thank you, the sentence has been amended thus:

Some GPs offer ‘bulk-billing’, a term which means they do not charge the patient a co-payment, rather Medicare covers the full consultation cost and patients authorise Medicare to pay the GP directly on their behalf.

8. **Bulk-billing at first mention (whether the table or text) needs explanation for international audiences. So too in the abstract or use a different term more widely understood (eg. Without a co-payment).**

We agree with the reviewer that it is difficult to explain bulk-billing as early as the abstract so have altered it to co-payment but we describe later in the manuscript the concept of bulk-billing as per the sentence above.

9. **In the sentence “Medication prescribed in general practice is typically issued as a private prescription, and the caregivers are responsible for the costs.” This needs to be qualified that this is medications for childhood gastroenteritis not medicines in general.**

We added ‘for childhood gastroenteritis’ in the sentence (Page 13, line 223).

10. **Re the sentence “For clinical care, the availability of medical equipment for the management of childhood gastroenteritis in primary care is minimal in both countries.” This still needs justification/clarification. Diagnostics and treatments are available in primary care for GE, but unlike in hospital settings, requires attending a second service provider. In addition, while point of care testing (presumably for electrolyte disturbances?) is generally not available in primary care, these are available in some remote/very remote primary care settings in Australia.**

We added ‘Primary care is not typically set up to monitor vital signs in an ongoing way or give fluids other than orally’ (Page 14, line 251-252)

11. **With respect to R1 Q3: I agree providing figures would help elucidate the point by giving a quantum reference for comparison (both for within each country as well between the countries).**

As reviewer 1 is very happy with the revised manuscript we are not sure what reviewer 2 wishes us to change here and we are happy we satisfied the comments made by reviewer 1.

12. **In the sentence “The Australian system acknowledges the various ways in which unscheduled care may be accessed, providing caregivers with options beyond the traditional GP visit,” what is meant by ‘traditional’ is unclear. Please revise.**

We have removed the word ‘traditional’.

13. **The paper should have general review for typographic and grammatical errors.**

We regret typographic and grammatical errors. We re-read the manuscript and corrected were appropriate. We would like to notice that we have two native speakers as co-author who checked the manuscript.
